# 

**Published:** 1891-12

**Authors:** 


### The Library of the Bristol Medico-Chirurgical Society.

---

At the Annual Meeting of this Society, held on October 14th, Mr. L. M. Griffiths, the Honorary Librarian, read the following Report:

It is only rarely that pluralism in the matter of offices is defensible: but in the case of this Society, where the connection between the *Journal* and the Library is of a very intimate nature, it will probably always be desirable that the Assistant-Editor of your *Journal*, or he who performs certain of the functions now appertaining to that office, should also be your Librarian.

A Librarian in his first Report is placed in a somewhat awkward position. It would hardly be appropriate that he should trace the history of libraries in general, or of special libraries from their first foundation; but, on the other hand, something more seems required than a mere enumeration of the volumes of which the Library consists, and a few words will certainly be in place recording the way in which this particular Library came into existence.

The close relationship of the *Journal* and Library to which I alluded supplies the explanation of the formation of our Library. When, over eight years ago, you decided to establish, more especially for the West of England and South Wales, a medical periodical which should in some worthy measure chronicle the excellent work done in these important districts, you took a step towards consolidating the professional interests of the neighbourhood, the importance of which it is impossible to exactly gauge. It was only fitting that a Society which, in addition to its own special functions, had thus done more to develop the professional life around it than had been done by any other of our medical institutions, should follow up its vigorous and public-spirited action by forming a library which should not only be a material help to its members in their daily work, but which might, in a certain sense, become the professional home of the medical men practising in its vicinity. This, after a few years, was rendered comparatively easy by the marked success of your *Journal*, which, under the guidance of its accomplished editor, has attained results almost, if not quite, phenomenal; and we, the members of this Society, have now the privilege, perhaps unique amongst medical societies in this world, at the cost of a half-guinea subscription, of not only obtaining the advantage which accrues from the attendance at meetings where the best work of highly-trained scientific and practical physicians and surgeons is brought forward, but also of receiving once a quarter a medical *Journal*, and, in addition, of enjoying freedom of access to a library well stored with current professional literature, and with a collection of books useful to the student and to the hard-working practitioner.

To those of us who have not yet passed the half-century, a medical library in Bristol is such a novelty that it will probably take us some little time to know how to use and value it properly; and we naturally look to the more experienced of the literary men around us to help us to the large extent which is in their power.

The Library is the direct outcome of the success of the *Journal*; and if it is to increase its efficiency, it must do so to a very large extent by the members of the Society extending in every possible way the circulation of the *Journal*, and by their own contributions raising more and more the literary standard of the publication. The Library ought also to react favourably on the roll of membership of the Society, for many should be attracted by the advantages it now offers.

In the summer of 1890, following upon a resolution which the Society passed pledging itself to formulate a scheme for the forma-tion of a Medical Library, the Committee received a request from an influential and considerable number of members, asking that the Library might, if only in a small way, be begun at once. This the Committee was only too glad to do, and the establishment of a Literary and Philosophic Club—one of the main purposes of which was to gather under one roof the scientific societies of Bristol—afforded, at a moderate outlay, the seasonable opportunity of pro-curing a suitable *habitat* for that for which we were seeking; and on January 5th, 1891, Mr. S. H. Swayne, the President of the Society opened, in a well-appointed room and amid congenial surroundings, the Library of the Bristol Medico-Chirurgical Society.

Several members of the Society took up the matter with enthu-siasm, and, by the aid of their generous donations, an auspicious beginning was made. The Committee of the Society supplied from the *Journal* reserve a handsome contribution of money—although it would be well not to draw too largely on this reserve, as a sub-stantial sum should be kept in hand wherewith to meet any emergency which might overtake the *Journal*, or by which further expensive developments of it might be possible.

The Committee decided that all periodicals, some 80 in number, and all books received by the *Journal* for review, should go to the Library, and thus, without any very considerable outlay, a fair show was made. Many important and useful gifts were received from members and others soon after the opening of the Library. These gifts required considerable additions to the shelf accommodation, and now members have at their command a collection of between 1,800 and 1,900 volumes.

A list of these books, arranged under the alphabetical order of their authors' names, has been made, and was printed in the last issue of the *Journal*. Mr. Harsant, one of the associate-editors of the *Journal*, and I have struggled to make this list typographically correct; but doubtless there are in it some errors, and we shall be grateful to anyone who will be kind enough to point them out to us. I hope to find an early opportunity to classify the books in the Library under their subjects, and so make the Library more useful to the seeker in a hurry.

It would be obviously unfair to compare our small Library with any of the magnificent collections so well known, and which are the result of long years of collecting, aided by munificent donations or bequests, or by national subsidy. But we have the potentialities of greatness, and it is given to every one to help to some extent. It is only natural that each memher of the Society should take a pride in this—his—Library, and it is to be expected that he will do everything that in him lies to promote its efficiency by making it as complete as possible. Many who are unable to do anything considerable in a direction such as this are often timid about offering a small contribution; but I hope that no member of the Society will feel any hesitation on this score. Many sets require volumes for their completion, and a visit to a second-hand bookseller might nearly always result in bringing away at a very low price something which would be a welcome addition to a medical library. If it were not a book in frequent request, it might take a place among other top-shelf literature, to which an occasional student is glad to turn.

Owing to the good fortune of the Society in obtaining a room in the Club House of the Literary and Philosophic Club, our Library is open from 10 a.m. to 11 p.m. This is an advantage which those who find it most convenient to pay a visit to the Library at a late hour in the day will thoroughly appreciate, and is one which I am not sure that any other medical library is able to offer.

Some may think it desirable that provision should be made for taking books out; but although this would undoubtedly be a convenience to the few, it would be a serious inconvenience to the many, for it is a great disappointment and worry to make a special journey to a library for the purpose of looking up some particular point and then to find that the book we wish to see is not there. In a library like ours the circulation of books could only be satisfactorily managed by a rigid system of pains and penalties, the enforcement of which would be both irksome and painful; and the need does not, in the case of books we wish to consult, exist to the same extent it does in many other branches of literature. But there can scarcely be an objection to the loan of duplicate books, and it will give me great pleasure to prepare a list of those that we have, and to make arrangements for members to take them home.

Some simple by-laws for the regulation of the Library have been drawn up, and will be found on the notice-board in the room and in the circular convening this meeting. These explain themselves; but in the interest of the frequenters of the Library generally, I should like to call special attention to No. 5, which beseeches members to put back books and periodicals in the places from which they took them. Omission to do this causes much unnecessary inconvenience to other readers, and to the Librarian when making up sets of periodicals for the binder.

The function of a library is not only to supply a demand, but to create a desire, and in both these directions it is not too much to expect that this recent departure of the Society will confer a boon much valued by the local profession. The noteworthy productions of the leaders of the profession teem with references to the work of their predecessors, by which an accepted theory or a plan of treatment or the perfection of an operation has been attained, and it is no slight advantage to be able on the spot to turn to these contributions. As all the books in the Library have passed through my hands, I may possibly know something more about our possessions than some of those who may seek the help to be afforded by them, and I shall be glad to impart any information I can, either by correspondence or by appointment at any time the enquirer may find convenient.

---

*The following donations have been received since the publication of the List in September:*

*November 30th*, 1891.

| | £ | s. | d. | | |
|---|---|---|---|---|---|
| Edmund C. Board ... ... ... | 5 | 5 | 0 | | |
| G. G. Corbould (1) ... ... ... | | | | ... 45 | volumes. |
| D. S. Davies, M.B. (2) ... ... | | | | ... 1 | volume. |
| James Fawn (3) ... ... ... | | | | ... 9 | volumes. |
| J. H. Norman (4) ... ... ... | | | | ... 2 | ,, |
| R. Shingleton Smith, M.D. (5) | | | | ... 10 | ,, |
| Simeon Snell (6) ... ... ... | | | | ... 1 | volume. |
| Surgeon-General U.S. Army (7) | | | | ... 1 | ,, |
| S. H. Swayne (8) ... ... ... | 5 | 0 | 0 | and 31 | volumes. |
| E. G. Tyrrell (9) ... ... ... | | | | ... 1 | volume. |

Unbound periodicals have been received from Mr. L. M. Griffiths and Mr. Norman.

Framed pictures have been presented by Mr. F. R. Cross, Mr. F. P. Lansdown, Miss Ethel Martyn, and Dr. J. G. Swayne.

---

### SECOND LIST OF BOOKS.

The figures in brackets refer to the figures after the names of the donors, and show by whom the volumes were presented. The books to which no such figures are attached have either been bought from the Library Fund or received through the *Journal*.

---

| | | | |
|---|---|---|---|
| **Baynton, T.** ... | *New Method of Treating Ulcers* ... ... ... ... ... | (3) | 1797 |
| **Bird, G.** ... ... | *Urinary Deposits* ... ... ... ... ... ... ... | 2nd Ed. | 1846 |
| **Boenning, H. C** | *Text-Book of Practical Anatomy* ... ... ... ... | | 1891 |
| **Bourke, Capt. J. G.** | *Scatalogic Rites of All Nations* .. ... ... .. ... | | 1891 |
| **Bramwell, B.** ... | *Atlas of Clinical Medicine.* Vol. I. Part II... ... | | 1891 |

**Budd, W.** ... *Malignant Pustule* ... ... ... ... ... ... (3) 1863
,, ... *Variola Ovina* ... ... ... ... ... ... ... (3) 1863

*Catalogue (Index) of Library of Surgeon-General's Office, U.S. Army* Vol. XII. ... ... ... ... ... ... ... ... (7) 1891
**Children, J. G.** *Chemical Analysis* ... ... ... ... ... ... (8) 1819
**Cloquet, H.** ... *Anatomy.* (Tr. by R. Knox). ... ... ... 2nd Ed. 1831
**Coombe, R.** ... *Pocket Pharmacopœia* ... ... ... ... ... ... ... 1891
**Cooper, Sir A.** ... *On the Testis.* 2nd Ed. (Ed. by B. B. Cooper)... 1841
,, ... *Dislocations and Fractures.* (Ed. by B. B. Cooper) 1842
**Coupland, S.** ... *Notes on the Examination of the Sputum, Vomit, Fæces, Urine, and Blood* ... ... ... ... 2nd Ed. 1891

**Doudney, G. H.** *The Water Cure in the Bedroom*... ... ... ... ... [1891]

**Forbes, J.** ... *Mesmerism True—Mesmerism False* ... ... ... (3) 1845

**Godfrey, Rev. N. S.** *Table-Turning* ... ... ... ... ... (3) 3rd Ed. 1853

**Haller, A.** ... *Physiology.* 2 vols. ... ... ... ... (8) 2nd Ed. 1772

**Hunter, J.** ... *Works.* (Ed. by J. F. Palmer.) 5 vols. Letter-press and Plates ... ... ... ... ... ... 1835–37

**James, Rev. W.** *Memoir of John Bishop Estlin* ... ... ... ... (3) 1855
**Jones, H. M.** ... *Diseases of Women and Uterine Therapeutics.* 5th Ed. 1891

**Kerr, N.** ... ... *Inebriety* ... ... ... ... ... ... (5) 2nd Ed. 1889

**Larkin and R. Leigh, F. C.** *Outlines of Practical Physiological Chemistry* ... ... ... ... ... ... ... ... 2nd Ed. 1891
**Latham, P. M.** .. *Diseases of the Heart.* Vol. I. ... ... ... 2nd Ed. 1846
**Leigh, F. C. Larkin and R.** *Outlines of Practical Physiological Chemistry* ... .. ... ... ... ... ... ... 2nd Ed. 1891

**Martyn, S.** ... *Hospitals, Past and Present* ... ... ... ... (3) 1862
**Miller, J.** ... ... *Principles of Surgery* ... ... ... ... ... ... ... 1844
**Murray, J.** ... *A System of Chemistry.* 4 vols. ... ... ... (8) 1806–7
**Muskett, P. E.** *Prescribing and Treatment for Infants and Children*... 1891

**Nicholson, H. A.** *Syllabus of Lectures on Zoology, &c.* ... ... ... (3) 1870
**Nissen, H.** ... *A B C of Swedish Educational Gymnastics* ... ... 1891

**Parkes, S.** ... *The Chemical Catechism* ... ... ... (8) 3rd Ed. 1808
**P[richard], A.** ... *Crosby Leonard* ... ... ... ... ... ... ... (3) [N.D.]
**Prout, W.** ... *On Stomach and Renal Diseases* ... ... 5th Ed. 1848

| | | | |
|---|---|---|---|
| **Remondino, P. C.** | *Circumcision* ... ... ... ... ... ... ... ... ... | | 1891 |
| **Rigby, E.** ... | *Midwifery* ... ... ... ... ... ... ... ... ... ... ... | | 1844 |
| **Roose, R.** ... ... | *Nerve Prostration* ... ... ... ... ... ... | 2nd Ed. | 1821 |
| **Savill, T.** ... ... | *Prescriber's Companion* ... .. ... ... ... | 2nd Ed. | 1891 |
| **Scott, B.** ... ... | *A State Iniquity* ... ... ... ... ... ... ... ... ... | (9) | 1890 |
| **Smith, R. W.** ... | *A Treatise on Fractures* ... ... ... ... ... ... ... | | 1850 |
| **Snell, S.** ... ... | *The Electro-Magnet and its Employment in Ophthalmic Surgery* ... ... ... ... ... ... ... ... ... | (6) | 1883 |
| *Surgical Practice, Golden Rules of* | ... ... ... ... ... ... ... ... ... | | [N.D.] |
| **Swieten, Baron v.** | *Commentaries upon Boerhaave's Aphorisms.* 18 vols.. ... ... ... ... ... ... ... ... | (8) | 1776 |
| **Thin, G.** ... ... | *Leprosy* ... ... ... ... ... ... ... ... ... ... ... ... | | 1891 |
| **Thomson, T.** ... | *A System of Chemistry.* 4 vols. ... | (8) 5th Ed. | 1817 |
| **Ure, A.** ... ... | *Dictionary of Chemistry* ... ... ... ... ... ... | (8) | 1821 |

---

### TRANSACTIONS, REPORTS, JOURNALS, &c.

*The List of current Periodicals to be seen in the Library is given in the* Journal *for March.*

| | |
|---|---|
| Hospital Annual, Burdett's, 1891-92... ... ... ... ... ... ... ... | |
| International Clinics. Vol. II. ... ... ... ... ... ... ... ... ... | 1891 |
| Leeward Islands Medical Journal ... ... ... ... ... ... ... ... | 1891 |
| Medical Chronicle, The Vol. XIV. ... ... ... ... ... ... ... ... | 1891 |
| Medical Record, The Vol. XXXIX. ... ... ... ... ... ... ... | 1891 |
| New York Academy of Medicine, Transactions of the Vol. VII. | 1891 |
| Retrospect of Medicine, Braithwaite's. (1) Vol. I., 3rd Ed., 1842; (1) Vol. II., 2nd Ed., 1842; (1) Vol. V., 1842; (1) Vols. IX., X., 1844-45; (1) Vol. XVI., 1847; (5) Vols. XXXVI.—XXXVIII., 1858-59; (1, 5) Vol. XXXIX., 1859; (5) Vol. XL., 1860; (1) Vols. XLI.—LI., 1860-65; (1) Vols. LIII.—LXV., 1866-72; (1) Vols. LXVII., LXVIII., 1873-74; (1) Vols. LXX.—LXXVI., 1874-78; (1) Vols. LXXVIII., LXXIX., 1879; (1) Vols. LXXXI.—LXXXIII. ... ... ... | 1880-81 |
| St. Andrew's Medical Graduates' Association, Transactions of the Vols. I.—IV. ... ... ... ... ... ... ... ... ... (5) | 1868-71 |
| Somerset, Summary of the Reports of the Medical Officers of Health for the County of ... ... ... ... ... ... ... ... (2) | 1891 |
| Year-Book of Treatment, The ... ... ... ... ... (4) 1885; (4) | 1886 |

### LIST OF SUBSCRIBERS

TO

### The Bristol Medico-Chirurgical Journal.

*DECEMBER*, 1891.

---

### England.

### BERKSHIRE.

MAIDENHEAD.
J. Lidderdale

### CHESHIRE.

CHESTER.
R. E. Burges

EGREMONT.
T. W. A. Napier

### CORNWALL.

BODMIN.
W. Pearse

LOSTWITHIEL.
J. H. Sewart

NEW QUAY.
A. Hardwick

PADSTOW.
H. F. Marley

PENZANCE.
W. S. Bennett
J. A. Fox

POLRUAN.
W. H. Torbock

REDRUTH.
J. S. Hichens

ST. AUSTELL.
W. Mason

ST. JUST.
R. B. Searle

TRURO.

J. B. Jackson

E. Rundle

### DEVONSHIRE.

BAMPTON.
T. A. Guinness

BARNSTAPLE.
C. H. Gamble
J. Harper
W. A. G. Laing
F. Penny

BRIXHAM.
G. C. Searle

BROADCLYST.
J. Somer

BUDLEIGH SALTERTON.
H. F. Semple

CHAGFORD.
A. D. Hunt

CHUDLEIGH.
C. H. Wade

DAWLISH.
A. de W. Baker
F. M. Cann

DEVONPORT.
J. Harrison
G. T. Rolston
F. E. Row

EXETER.
J. M. Ackland
A. G. Blomfield
H. Davy
P. M. Deas
E. J. Domville
W. Gordon
A. W. Kempe
E. B. Steele-Perkins
L. H. Tosswill

HONITON.
T. W. Shortridge

LYNTON.
F. C. Berry

MODBURY.
J. H. Harris

NEWTON ABBOT.
E. Haydon

OTTERY ST. MARY.
F. A. Gray

PAIGNTON.

J. Alexander

R. H. Norgate

### DEVONSHIRE *(Continued)*.

PLYMOUTH.
R. H. Clay
E. E. Meeres
W. G. Nash
E. B. Thomson
W. L. Woollcombe

PLYMPTON.
W. D. Stamp

ST. MARY CHURCH.
T. Finch

SALCOMBE.
A. H. Twining

SOUTH MOLTON.
E. Furse

TAVISTOCK.
M. T. Leamon

TIVERTON.
J. S. Smith

TORQUAY
G. F. P. Pizey
R. Pollard
W. Powell

UFFCULME.
F. Morgan

WINKLEIGH.
J. H. Norman

YELVERTON.
A. P. Prowse

### DORSETSHIRE.

BEAMINSTER.
T. P. Daniel
F. P. Kitson

DORCHESTER.
F. B. Fisher
P. W. Macdonald

PARKSTONE.
W. H. Masters

POOLE.
A. C. Kemble
J. McNicoll

PORTLAND.
G. H. Lilley

SHERBORNE.
T. R. Atkinson
W. H. Williams

STURMINSTER NEWTON.
J. C. Leach

WEYMOUTH.
R. W. Carter
J. M. Lawrie
W. G. V. Lush

WIMBORNE.
A. J. H. Crespi

### ESSEX.

NEWPORT.
W. A. Smith

SOUTHEND.
E. E. Phillips

### GLOUCESTERSHIRE.

BRISTOL.

W. R. Ackland
G. Adams
J. B. Adams
G. F. Atchley
W. M. Barclay
T. G. L. Baretti
B. J. Baron
A. E. Blacker
A. F. Blagg
E. C. Board
J. G. Boyce
J. Broom
G. F. Burder
E. F. H. Burroughs
J. P. Bush
F. Calder
J. Cameron
A. Carr
W. F. Carter
J. M. Clarke
E. W. Coathupe
R. W. Coe
T. V. Coker
T. J. Colman
H. Cook
G. G. Corbould
W. Cotton
F. R. Cross
J. Dacre
D. S. Davies
H. F. Devis
H. R. Dew
N. C. Dobson
W. Dowson
E. L. W. Dunbar
F. H. Edgeworth
W. Elder
C. Elliott
W. R. Etches
J. Ewens
R. G. Fendick
E. L. Fox
W. J. Fyffe
G. Gardiner
A. N. G. Gibbs
J. Gill
G. A. Gloag
W. C. Goodden
W. G. Grace
L. M. Griffiths
C. D. G. Hailes
A. H. Haines
A. J. Harrison
W. H. Harsant
A. G. Hayman
J. C. Heaven
C. K. C. Herapath
H. E. Hetling
F. F. Hills
A. W. W. Hoffman
General Hospital
G. A. Imlay
Royal Infirmary
J. Irving
D. G. Johnston
F. P. Lansdown
R. G. P. Lansdown
A. E. A. Lawrence
H. Lawrence
E. L. Lees
Museum and Library
W. E. Lloyd
F. T. B. Logan
E. J. Maclean
W. T. Maddison
H. Marshall
C. E. Matthews
T. O. Mayor
J. S. Metford
J. Milne
H. F. Mole
C. A. Morton
B. G. Neale
W. N. Nevill
C. Newman
W. H. C. Newnham
J. Newstead
T. D. Nicholson
J. A. Norton
A. Ogilvy
G. S. Page
G. Parker
J. H. Parry
T. C. Parson
G. C. P. Pauli
W. J. Penny
J. H. Perkins
C. F. Pickering
W. E. Pountney
J. J. Powell
A. Prichard
A. W. Prichard
J. E. Prichard
A. Pring

### GLOUCESTERSHIRE (*Continued*).

BRISTOL (*continued*).

A. B. Prowse
B. M. H. Rogers
W. B. Roué
C. K. Rudge
H. T. Rudge
J. E. Shaw
E. J. Sheppard
W. E. Shorland
E. M. Skerritt
G. M. Smith
J. G. Smith
R. S. Smith
S. Smith
R. V. Solly
A. A. Sproule
G. M. Stansfeld
C. Steele
W. H. Stevens
F. W. Stoddart
J. Swain
J. G. Swayne
S. H. Swayne
W. C. Swayne
J. Taylor
R. W. Thomas
W. J. Tivy
H. Waldo
C. H. Walker
C. H. Wallace
J. H. Wathen
T. Webster
C. E. Wedmore
J. Wilding
P. W. Williams
H. W. Windsor-Aubrey

CHALFORD.

F. C. Palmer

CHELTENHAM.

Cheltenham Library
C. J. Newton
F. A. A. Smith
L. Winterbotham

CHIPPING SODBURY.

A. Grace

CINDERFORD.

W. C. Heane

COLEFORD.

L. B. Trotter

DOWNEND.

H. Skelton

FISHPONDS.

W. Brown

GLOUCESTER.

R. W. Batten
T. Edis
T. S. Ellis

HAMBROOK.

E. Crossman
W. F. B. Eadon

HAWKESBURY-UPTON.

W. I. Cox

KINGSWOOD.

H. Grace

MORETON-IN-MARSH.

L. K. Yelf

OLVESTON.

N. H. Lower
E. H. Openshaw

STAPLE HILL.

W. Murray

STAPLETON.

H. A. Benham

STOKE BISHOP.

H. Appleton
J. Stewart

STONEHOUSE.

G. T. B.Watters

STOW-ON-THE-WOLD.

E. Dening

STROUD.

A. S. Cooke
E. A. Howsin
F. W. Storry

TETBURY.

W. Wickham

THORNBURY.

E. M. Grace
T. H. Taylor
L. H. Williams

WESTBURY-ON-TRYM.

W. Duncan
H. Ormerod

WINTERBOURNE.

R. Eager

WOTTON-UNDER-EDGE.

D. H. Forty

### HAMPSHIRE.

BOURNEMOUTH.

D. C. Embleton
J. Horsfall
S. T. Smyth

CARISBROOKE.

J. Groves.

EMSWORTH.

L. Stephens

LYMINGTON.

J. Rendall

ST. MARY BOURNE.

A. W. Riddell

SOUTHSEA.

J. W. Cousins

VENTNOR.

R. Robertson

### HEREFORDSHIRE.

HEREFORD.

W. C. Whitfeld

### HERTFORDSHIRE.

ST. ALBANS.

A. H. Boys

### KENT.

DARTFORD.

A. H. Syrée

TUNBRIDGE WELLS.

C. Wilson

### LANCASHIRE.

LIVERPOOL.

Medical Institution

POULTON-LE-FYLDE.

P. H. Day

### MIDDLESEX.

LONDON.

J. Abercombie
L. S. Beale
J. Berry
L. Browne
T. Buzzard
J. G. Davey
J. F. Evans
A. P. Gould
J. M. Granville
M. Grigor
M. Handfield-Jones
W. P. Herringham
W. H. A. Jacobson
C. M. Jessop
J. Lister
J. Macready
A. L. Marshall
W. Pippette
R. D. Powell
M. A. Ruffer
W. J. Seward
N. Smith
Royal Med. and Chir. Society
J. A. Watson
F. J. Wethered

TOTTENHAM.

W. H. Plaister

### MONMOUTHSHIRE.

NEWPORT.

W. Basset
C. B. Gratte
H. R. Hudson
T. Limbery
O. E. B. Marsh
A. G. Thomas
J. T. Thomas
H. E. Williams

PONTYPOOL.

S. B. Mason

RHYMNEY.

T. H. Redwood

RISCA.

G. B. Robathan

TREDEGAR.

G. A. Brown

### NOTTINGHAMSHIRE.

NOTTINGHAM.

C. B. Taylor

### OXFORDSHIRE.

OXFORD.

R. W. Doyne
H. P. Symonds

### SOMERSETSHIRE.

BATH.

H. T. S. Aveline
G. A. Bannatyne
G. E. Bloxam
A. B. Brabazon
T. Cole
F. S. Cowan
S. Craddock
R. Davis
W. M. Ellis
A. E. W. Fox
H. W. Freeman
H. F. A. Goodridge
T. B. Goss
F. P. Hoblyn
H. C. Hopkins
L. J. King
F. Lace
H. Lane
T. P. Lowe
R. J. H. Scott
E. Skeate
J. K. Spender
L. A. Weatherly

BECKINGTON.

W. G. Evans

BRIDGWATER.

T. L. Laxton
F. J. C. Parsons
J. B. Sincock

BRISLINGTON.

B. B. Fox
C. H. Fox

BURNHAM.

A. W. C. Peskett

CHEDDAR.

R. W. Statham

CHEW MAGNA.

E. T. Hale

CLEVEDON.

T. Davis
J. A. F. Sawyer
S. Skinner

CREWKERNE.

E. J. Cave
W. W. Webber

### SOMERSETSHIRE *(Continued).*

DULVERTON.
G. F. Sydenham

FRESHFORD.
C. E. S. Flemming

FROME.
E. Cockey
F. Parsons
J. M. Rattray

GLASTONBURY.
J. A. Bright

HIGHBRIDGE.
R. Wade

HUNTSPILL.
J. H. Sharpe

ILMINSTER.
C. Munden

KEYNSHAM.
G. G. D. Willett

LANGPORT.
J. Morgan

MIDSOMER NORTON
E. Blacker
A. Waugh

MILVERTON.
C. Randolph

MINEHEAD.
T. Clark
T. J. Ollerhead

NORTH PETHERTON.
C. F. Hawkins

PILL.
R. A. Ross

PORTISHEAD.
W. Monckton
C. A. Wigan

SOUTH PETHERTON.
W. H. Walter

STREET.
A. Clarke

TAUNTON.
H. Alford
H. J. Alford
Taunton Hospital
W. Liddon

TEMPLE CLOUD.
T. Martin

TWERTON-ON-AVON.
J. Wigmore

WEDMORE.
J. Ford
R. P. Tyley

WELLINGTON.
J. Meredith
T. E. P. Prideaux

WELLS.
F. J. B. Bateman
W. Fairbanks
A. L. Wade

WESTON-SUPER-MARE.
G. E. Alford
C. P. Crouch
G. B. Fraser
E. F. Martin
W. H. G. Phelps
G. F. Rossiter
R. Roxburgh

WRINGTON.
A. H. Benson
H. W. Collins

YEOVIL.
P. S. H. Colmer
C. J. Marsh
E. Vernon

### STAFFORDSHIRE.

STAFFORD.
J. Scott

### SURREY.

CROYDON.
R. R. Whishaw

EPSOM.
R. Davis

ESHER.
J. B. Fry

### SUSSEX.

BRIGHTON.
H. H. Tomkins

ST. LEONARD'S-ON-SEA.
W. H. Spencer

### WARWICKSHIRE.

BIRMINGHAM.
L. Tait

### WILTSHIRE.

BRADFORD-ON-AVON.
W. J. A. Adye
J. Beddoe

BURBAGE.
A. E. N. Browne

CALNE.
W. S. Batten

DEVIZES.

T. B. Anstie

C. Eddowes

FOVANT.
C. Clay

MALMESBURY.
R. Kinneir

SALISBURY.
L. S. Luckham
H. J. Manning

SWINDON.

E. D. Duffett

H. W. M. Hayes

W. Howse

J. C. Maclean

WARMINSTER.
J. Hinton
R. L. Willcox

### WORCESTERSHIRE.

WORCESTER.
J. F. Royle

### YORKSHIRE.

HARROGATE.
A. O. Jones

HULL.
J. B. Close

SHEFFIELD.
S. Snell

### Ireland.

### ANTRIM.

BELFAST.

J. W. Browne

### LOUTH.

DUNDALK.

J. Browne

### Scotland.

### EDINBURGHSHIRE.

EDINBURGH.

J. W. Ballantyne
Y. J. Pentland

### Wales.

### BRECONSHIRE.

BRECON.

A. Whyte

BRYNMAWR.

G. H. Browne

SENNY BRIDGE.

W. R. Jones

TALGARTH.

W. Howells

### CARDIGANSHIRE.

LAMPETER.

E. C. Davies

NEW QUAY.

E. R. Jones

### CARMARTHENSHIRE.

CARMARTHEN.

G. J. Hearder
H. Hill

FERRYSIDE.

P. Williams

LLANELLY.

D. J. Williams

### GLAMORGANSHIRE.

ABERAMAN.

J. B. James

ABERDARE.

E. Jones

CAERPHILLY.

J. Lewellyn

CARDIFF.

W. T. Edwards
G. W. Grote
J. Milward
D. R. Paterson
W. Price
R. Prichard
A. Sheen
Medical Society
J. T. Thompson
C. T. Vachell
H. R. Vachell
C. Williams

DOWLAIS.

E. S. S. Jones

LLANDAFF.

J. Arthur

MERTHYR TYDVIL.

J. L. W. Ward

PENARTH.

W. J. Clapp
W. A. Moynan

PORTH.

H. N. Davies

REYNOLDSTONE.

H. V. Ellis

SWANSEA.

E. Davies
T. C. Grey
G. Padley
J. Thomas

TREHARRIS.

W. W. Leigh

### PEMBROKESHIRE.

TENBY.

B. Kendall

### Channel Islands.

GUERNSEY.

N. Webster

### Belgium.

COURTRAI.

Dr. Lauwers

### Greece.

CORFU.

Dr. Mavrojanni

### Italy.

PALERMO.

C. Scimonelli

### Russia.

ODESSA.

G. Schleicher

### Switzerland.

DAVOS-PLATZ.

W. R. Huggard

### Algeria.

ALGIERS.

W. Thomson

### Cape Colony.

FORT BEAUFORT.

J. Shaw

### INDEX.

Abortion, 108.
Albuminuria, Intermittent, 278.
Allan, Dr. F. J.—Aids to sanitary science (*Review*), 57.
Anæsthetics, 290.
Annual of the universal medical sciences (*Review*), 305.
Antiseptics in midwifery, 105, 292.
Atlas of clinical medicine—Dr. B. Bramwell (*Review*), 205, 307.
Aural surgery, Dangers of, 117, 118.

Baby, Our—Mrs. L. Hewer (*Review*), 134.
Bacillus, Tubercle, 96.
Bacteria and their products—Dr. G. S. Woodhead (*Review*), 302.
Bacteriology, 281.
Bacteriology, Manual of—Dr. E. M. Crookshank (*Review*), 121.
Ball, Dr. J. B.—Diseases of nose (*Review*), 56; Intubation of the larynx (*Review*), 210.
Ballantyne, Dr. J. W.—Diseases of infancy (*Review*), 204.
Bannatyne, Dr. G. A.—Epilepsy treated by sulphonal, 261.
Baron, Dr. B. J.—Reports on laryngology and rhinology, 112, 297.
Bell, Dr. J.—Notes on surgery for nurses (*Review*), 301.
Bennett, W. H.—On varicocele (*Review*), 209.
Berry, Dr. G. A.—Ophthalmoscopic diagnosis (*Review*), 132.
Bibliography, International medical, 307.
Bibliotheca medico-chirurgica (*Review*), 211.
Blood, Quality of the, 188.
Booksellers, Directory of second-hand (*Review*), 135.
Bowles, Dr. R. L.—Stertor, apoplexy, and the management of the apoplectic state (*Review*), 207.
Brain development, 47.
Bramwell, Dr. B.—Atlas of clinical medicine (*Review*), 205, 307.
Bristol Medico-Chirurgical Society, 63, 140, 311.
Brittan, Dr. F., 67.
Browne, L.—Diseases of throat and nose (*Review*), 56; Koch's remedy in throat consumption (*Review*), 126.
Bruce, Dr. J. M.—Materia medica (*Review*), 129.
Burnet, Dr. R. W.—Foods and dietaries (*Review*), 58.
Buxton—A. J. H. Crespi, 167.

Cancer as an infective disease, 185.
Cancer of uterus, 197.
Cantharidin, 115, 297.
Champneys, Dr. F. H.—On painful menstruation (*Review*), 208.
Chloroform *versus* ether — W. H. Harsant, 145.
Ciliary processes, Anatomy of, 198.
Clarke, Dr. J. M.—Diagnosis in gastric affections, 73; reports on medicine, 31, 94.
Colotomy, 39.
Coupland, Dr. S.—Notes on the examination of the sputum, vomit, fæces, and urine (*Review*), 134.
Craniectomy, 98.
Crespi, A. J. H.—Buxton, 167.
Crookshank, Dr. E. M.—Manual of bacteriology (*Review*), 121.
Cross, F. R.—Medical progress, local and general, 241; reports on ophthalmology, 40, 198.

Diabetes—Dr. C. W. Purdy (*Review*), 52; Dr. R. Saundby (*Review*), 128.
Diary, Letts's medical (*Review*), 305.
Digest, The medical—Dr. R. Neale (*Review*), 210.
Diphtheria, 115, 186, 298.
Duboisin, 48.

Ear, Diseases of—Dr. J. Gruber (*Review*), 54.
Edgeworth, Dr. F. H.—Septic endocarditis, 89.
Edinger, Dr. L.—Structure of central nervous system (*Review*), 51.
Electricity in diseases of women—Dr. G. B. Massey (*Review*), 59; 194.
Endocarditis, Septic—Dr. F. H. Edgeworth, 89.
Epilepsy—Dr. H. A. Hare (*Review*), 122.
Epilepsy treated by sulphonal—Dr. G. A. Bannatyne, 261.
Epithelioma of larynx, 116.
Examination of sputum, &c.—Dr. S. Coupland (*Review*), 134.
Eyeball, Wound of, 148.
Eye troubles associated with malarial poisoning, 47.

Felkin, Dr. R. W.—Hypnotism (*Review*), 50.
Fever—Dr. H. A. Hare (*Review*), 135.
Foods and dietaries—Dr. R. W. Burnet (*Review*), 58.
Furuncles of the auditory canal, 118.

Gallstones, 188.
Garrett, Dr. J. H.—The action of water on lead (*Review*), 127.
Gastric affections, Diagnosis in—Dr. J. M. Clarke, 73; 280.
Gerrard, A. W.—New official remedies, British Pharmacopœia, 1890 (*Review*), 208.
Gonorrhœa, Abortive treatment of, 288.
Griffiths, L. M.—Reports on obstetrics, 103, 292.
Gruber, Dr. J.—Diseases of ear (*Review*), 54.
Guy's hospital reports (*Review*), 301.
Gynæcology, Report on—Dr. W. H. C. Newnham, 194.

Hare, Dr. H. A.—Epilepsy (*Review*), 122; Fever (*Review*), 135.
Harsant, W. H.—Chloroform *versus* ether, 145; report on otology, 117; report on surgery, 286.
Headaches, Ocular, 45.
Heredity, health and personal beauty—Dr. J. V. Shoemaker (*Review*), 120.
Hewer, Mrs. L.—Our baby (*Review*), 134.
Hospital annual, Burdett's (*Review*), 304.
Hutchinson, P. S.—Diseases of nose and throat (*Review*), 136.
Hydrocele, 289.
Hygiene—Dr. G. H. Rohé (*Review*), 57; Dr. B. A. Whitelegge (*Review*), 57.
Hyoscyamine sulphate as a mydriatic, 48.
Hypnotism—Dr. R. W. Felkin (*Review*), 50.

Infancy, Diseases of—Dr. J. W. Ballantyne, (*Review*) 204; Dr. L. Starr (*Review*), 299.
Influenza, 47, 117, 118, 284.
Injection in eye diseases, 201.
International clinics (*Review*), 206.
Intubation of larynx—Dr. J. B. Ball (*Review*), 210.

Jones, Dr. H. M.—Subjective noises in the head and ears (*Review*), 300.

Koch's remedy in throat consumption—L. Browne (*Review*), 126.

Laboratory of the Royal College of Physicians of Edinburgh, Reports from (*Review*), 130.
Lane, H.—Rheumatic diseases (*Review*), 55.
Laryngology and rhinology, Reports on—Dr. B. J. Baron, 112, 297.
Lateral sinus, Septic thrombosis of—C. F. Pickering, 155.
Lead, Action of water on—Dr. J. H. Garrett (*Review*), 127.
Library of Bristol Medico-Chirurgical Society, 66, 215, 313.
Life insurance, Albuminuria and, 279.
Lunacy Act of 1890, 283.
Lung, Surgery of, 92.

M.D.—Rhyming and mnemonic key to materia medica (*Review*), 211.
Malvern—W. Tyrrell, 267.
Marston, Dr. J. A.—Notes on typhoid fever (*Review*), 121.
Massey, Dr. G. B.—Electricity in diseases of women (*Review*), 59.

Materia medica—Dr. J. M. Bruce (*Review*), 129; Dr. J. V. Shoemaker (*Review*), 51; M.D. (*Review*), 211.
Medical annual (*Review*), 125.
Medical progress, local and general —F. R. Cross, 241.
Medicine, Handbook of—Dr. F. T. Roberts (*Review*), 54.
Medicine, Practical — Dr. A. H. Carter (*Review*), 137.
Medicine, Reports on—Dr. J. M. Clarke, 31, 94; Dr. J. E. Shaw, 278; Dr. R. Shingleton Smith, 184, 284.
Menstruation, Painful—Dr. F. H. Champneys (*Review*), 208.
Mental derangements following operations on eye, 47.
Mind, Unsoundness of—J. W. H. Williams (*Review*), 53.
Moullin, C. W. M.—Surgery (*Review*), 123.

Naphthalin, 48.
Napier, Dr. A. D. L.—Thermometer in obstetrics (*Review*), 122.
Nasal reflexes, 115, 298.
Neale, Dr. R.—The medical digest (*Review*), 210.
Nerves, Operations on, 189.
Nervous system, Structure of central —Dr. L. Edinger (*Review*), 51.
Newnham, Dr. W. H. C.—Report on gynæcology, 194.
Noises in the head and ears—Dr. H. M. Jones (*Review*), 300.
Nose, Diseases of—Dr. J. B. Ball (*Review*), 56; L. Browne (*Review*), 56; P. S. Hutchinson (*Review*), 136; W. S. Watson (*Review*), 56.
Nurses, Surgery for—Dr. J. Bell (*Review*), 301.
Nystagmus, 202.

Obstetric curiosities, 109.
Obstetrics, Reports on—L. M. Griffiths, 103, 292.
Obstetrics, Training in, 111.
Ointments and oleates—Dr. J. V. Shoemaker (*Review*), 60.
Ophthalmia, Sympathetic, 45.
Ophthalmology, Reports on—F. R. Cross, 40, 198.
Ophthalmoscopic diagnosis—Dr. G. A. Berry (*Review*), 132.
Otology, Report on—W. H. Harsant, 117.

Pancreas in diabetes, Lesions of, 97.
Parkin bequest of Royal College of Physicians of Edinburgh, 70.
Parturition, Mechanical effects of, 104.
Penny, W. J.—Reports on surgery, 189, 291.
Pharmacopœia, Additions to—A. W. Gerrard (*Review*), 208; Dr. F. T. Roberts (*Review*), 208.
Philip, Dr. R. W—Pulmonary tuberculosis (*Review*), 133.
Pickering, C. F.—Cases of septic thrombosis of lateral sinus, 155.
Pilocarpine in deafness, 117.
Pons, Tumour of—Dr. P. W. Williams, 163.
Pregnancy, Duration of, 103.
Prichard, A. W.—Case of poisoning by rhus venenata, 22.
Prichard and Symonds—Dr. D. H. Tuke (*Review*), 306.
Prisms, 40.
Puerperal eclampsia — Dr. J. G. Swayne, 1; 106.
Pulmonary lesions in hereditary syphilis, 96.
Purdy, Dr. C. W.—Diabetes (*Review*), 52.
Pyoktanin, 116.

Retinal detachment, 45.
Rheumatic diseases—H. Lane (*Review*), 55.
Rhus venenata, Case of poisoning by—A. W. Prichard, 22.
Roberts, Dr. F. T.—Handbook of medicine (*Review*), 54; Notes on the additions made to the British Pharmacopœia, 1890, 208.
Rohé, Dr. G. H.—Hygiene (*Review*), 57.

Sanitary science, Aids to—Dr. F. J. Allan (*Review*), 57.
Saundby, Dr. R.—Diabetes (*Review*), 128.
Scraps, 71, 143, 238, 319.
Senn, Dr. N.—Principles of surgery (*Review*), 119.
Shaw, Dr. J. E.—Report on medicine, 278.

Shoemaker, Dr. J. V.—Heredity, health and personal beauty (*Review*), 120; Materia medica (*Review*), 51; Ointments and oleates (*Review*), 60.
Sick, Preparations for the—61, 137, 213, 308.
Sinuses, 291.
Smith, J. Greig—Reports on surgery, 35, 99.
Smith, Dr. R. Shingleton—Reports on medicine, 184, 284.
Starr, Dr. L.—Diseases of the digestive organs in infancy and childhood (*Review*), 299.
Stertor, apoplexy, &c,—Dr. R. L. Bowles (*Review*), 207.
Still-births, Life-saving methods in, 109.
St. Thomas' hospital reports (*Review*), 301.
Surgery—C. W. M. Moullin (*Review*), 123.
Surgery, Principles of—Dr. N. Senn (*Review*), 119.
Surgery, Reports on—W. H. Harsant, 286; W. J. Penny, 189, 291; J. Greig Smith, 35, 99.
Swayne, Dr. J. G. — Puerperal eclampsia, 1.
Symbolism, Medical — Dr. T. S. Sozinskey (*Review*), 212.
Symonds, Prichard and—Dr. D. H. Tuke (*Review*), 306.
Syphilis, Abortive treatment of, 286.

Thermometer in obstetrics—Dr. A. D. L. Napier (*Review*), 122.
Throat, Diseases of nose and—L. Browne (*Review*), 56; P. S. Hutchinson (*Review*), 136.
Trachoma, 46.
Tractor, The atmospheric, 105.
Tuberculin, 31, 37, 49, 94, 112, 126, 184, 283, 297.
Tuberculosis, Case of laryngeal—Dr. P. W. Williams, 27.
Tuberculosis, Pulmonary — Dr. R. W. Philip (*Review*), 133.
Tuke, Dr. D. H. — Prichard and Symonds in especial relation to mental science (*Review*), 306.
Typhoid fever, Notes on—Dr. J. A. Marston (*Review*), 121.
Tyrrell, W.—Malvern, 267.

Uterine appendages, Removal of—36.
Uterine diseases, Referred neuroses in, 196.

Varicocele—W. H. Bennett (*Review*), 209.
Virchow testimonial fund, 142.
Vermiform appendix, Inflammation of, 99.
Vocabulary, Lewis's pocket medical (*Review*), 212.

Watson, W. S. — Diseases of nose (*Review*), 56.
Whitelegge, Dr. B. A. — Hygiene (*Review*), 57.
Williams, J. W. H.—Unsoundness of mind (*Review*), 53.
Williams, Dr. P. W.—Case of laryngeal tuberculosis, 27; case of tumour of the pons, 163.
Woodhead, Dr. G. S.—Bacteria and their products (*Review*), 302.

Year-book of scientific societies (*Review*), 126.
Year-book of treatment (*Review*), 125.

### PUBLICATIONS RECEIVED.

From BAILLIÈRE, TINDALL & COX, London:
*Pocket Pharmacopœia.* By RUSSELL COOMBE. 1891.
*Diseases of Women.* By H. MACNAUGHTON JONES. Fifth Edition. 1891.
*The Medical Press and Circular.* Sept. 23, 1891.
*The Hospital Gazette.* November 21, 1891.
*Psycho-Therapeutics.* By C. LLOYD TUCKEY, M.D. Third Edition. 1891.
*The Human Ear.* By Dr. ADAM POLITZER. 1892.

From JOHN BALE & SONS, London:
*Prescriber's Companion.* By THOMAS SAVILL, M.D. Second Edition. 1891.

From BURROUGHS, WELLCOME & CO., London:
*Colour Test Chart.* By JOHN BROWN, M.D.

From EV. E. CARRERAS, St. Louis:
*The Alienist and Neurologist.* October, 1891.

From CASSELL & COMPANY LIMITED, London:
*Letts's Medical Diary for* 1892.
*First Lines in Midwifery.* By G. ERNEST HERMAN, M.B. 1891.
*A Manual of Operative Surgery.* By FREDERICK TREVES. 2 vols. 1891.

From J. & A. CHURCHILL, London:
*Leeward Islands Medical Journal.* Edited by H. A. ALFORD NICHOLLS, M.D. 1891.
*The Pathology of Bronchial Affections and Pneumonia.* By A. G. AULD, M.D. 1891.

From F. A. DAVIS, Philadelphia:
*Circumcision.* By P. C. REMONDINO, M.D. 1891.
*Text-Book of Practical Anatomy.* By HENRY C. BOENNING, M.D. 1891.
*The Medical Bulletin.* October, November, 1891.
*A B C of Swedish Educational Gymnastics.* By HARTVIG NISSEN. 1891.
*Age of the Domestic Animals.* By RUSH SHIPPEN HUIDEKOPER, M.D. 1891.

From GOVERNMENT PRINTING OFFICE, Washington:
*Index Catalogue of the Library of the Surgeon-General's Office, United States Army.* Vol. XII. REGER—SHUTTLEWORTH. 1891.

From *THE HOSPITAL* (LIMITED), London:
*Burdett's Hospital Annual.* 1891—1892.

From H. K. LEWIS, London:
*Nerve Prostration.* By ROBSON ROOSE, M.D. Second Edition. 1891.
*The British Journal of Dermatology.* October—December, 1891.
*Notes on the Examination of the Sputum, Vomit, Fæces, Urine, and Blood.* By SIDNEY COUPLAND, M.D. Second Edition. 1891.
*Outlines of Practical Physiological Chemistry.* By F. CHARLES LARKIN and RANDLE LEIGH, M.B. Second Edition. 1891.
*History of Medical Education.* By Dr. THEODOR PUSCHMANN. 1891.
*Inherited Consumption.* By WILLIAM DALE, M.D. 1891.

From LONGMANS, GREEN & CO., London:
*Epidemic Influenza.* By RICHARD SISLEY, M.D. 1891.

From W. H. LOWDERMILK & CO., Washington:
*Scatalogic Rites of all Nations.* By Captain JOHN G. BOURKE. 1891.

From MEDICAL HERALD CO., Saint Joseph, Mo.:
*The Medical Herald.* September, 1891.

From YOUNG J. PENTLAND, Edinburgh:
*Prescribing and Treatment for Infants and Children.* By PHILIP E. MUSKETT. 1891.
*International Clinics.* Edited by JOHN M. KEATING, M.D.; J. P. CROZIER GRIFFITH, M.D.; J. MITCHELL BRUCE, M.D.; DAVID W. FINLAY, M.D. Volume Second. 1891.

From PERCIVAL & CO., London:
*Leprosy.* By GEORGE THIN, M.D. 1891.

From JOHN MORGAN RICHARDS, London:
*Medical Reprints.* September—November, 1891.

From 30 RUE D'ASSAS, Paris:
Clinique Française. *Programme des Cours.* 1891.

From VANONI & CO., London:
*Vanoni's Weekly Sheet.* Nos. 1—4. 1891.

From THE VICTORIA STREET SOCIETY FOR THE PROTECTION OF ANIMALS FROM VIVISECTION, London, S.W.:
*A Foretaste of the Institute of Preventive Medicine.* 6.91
*An Institute of Preventive Medicine at Work in France.* Second Edition. July 1st, 1891.
*The Official Return for the Year ending December 31st,* 1891. 7.91.
*The Significance of Vivisection.* By FRANCES POWER COBBE. 7.91.
*Memorial to the Home Secretary.* November 3rd, 1891.

From JOHN WRIGHT & CO., Bristol:
*Golden Rules of Surgical Practice.* By A HOSPITAL SURGEON. [N.D.]
*The Water Cure in the Bedroom; or, Hydropathy at Home.* By G. H. DOUDNEY, M.B. [1891.]
*The Practice of Hypnotic Suggestion.* By GEO. C. KINGSBURY, M.D. 1891.

### Bristol Medico-Chirurgical Journal.

### DECEMBER, 1891.

### CONTENTS.

ORIGINAL PAPERS: *Page*

Medical Progress, Local and General. By F. R. CROSS, M.B. Lond., F.R.C.S. Eng. ... ... ... ... ... ... ... ... ... ... ... ... 241

Epilepsy Treated by Sulphonal. By GILBERT A. BANNATYNE, M.D., C.M. Glasg. ... ... ... ... ... ... ... ... ... ... ... ... 261

HEALTH-RESORTS IN THE WEST OF ENGLAND AND SOUTH WALES ... ... 267

PROGRESS OF THE MEDICAL SCIENCES ... ... ... ... ... ... ... ... ... 278

REVIEWS OF BOOKS ... ... ... ... ... ... ... ... ... ... ... ... ... ... 298

NOTES ON PREPARATIONS FOR THE SICK ... ... ... ... ... ... ... ... 309

MEETINGS ... ... ... ... ... ... ... ... ... ... ... ... ... ... ... ... 311

THE LIBRARY OF THE BRISTOL MEDICO-CHIRURGICAL SOCIETY ... ... ... 313

SCRAPS ... ... ... ... ... ... ... ... ... ... ... ... ... ... ... ... 319

*Cases for binding, price 7d. each, may be had of the Publisher,* J. W. ARROWSMITH, *Quay Street, Bristol.*

